# Misdiagnosis of spinocerebellar ataxia type 3 as persistent postural-perceptual dizziness: A case report

**DOI:** 10.1097/MD.0000000000041850

**Published:** 2025-04-04

**Authors:** Yisi Zhang, Xiaoguang Chen, Songbin Pan

**Affiliations:** a Department of Neurology, Traditional Chinese and Western Medicine Hospital of Wuhan, Tongji Medical College, Huazhong University of Science and Technology, Wuhan, China; b Department of Neurology, Wuhan No. 1 Hospital, Wuhan, China.

**Keywords:** SCA3, vestibular dyfunction, video head impulse test

## Abstract

**Rationale:**

Spinocerebellar ataxia type 3 (SCA3) is the most common autosomal dominant inherited spinocerebellar disorder. The disease can easily be misdiagnosed because the symptoms of SCA3 are diverse and not obvious in the early stages.

**Patient concerns:**

A 55-year-old woman had experienced dizziness and an unstable gait since 2017. She was misdiagnosed with persistent postural-perceptual dizziness at another hospital because of normal brain magnetic resonance imaging and emotional abnormalities. In our hospital, there were no abnormalities in routine laboratory tests and brain magnetic resonance imaging, but various examinations showed peripheral and central vestibular dysfunction. She had a typical family history of dizziness and an unstable gait.

**Diagnoses:**

The patient was diagnosed with SCA3 by genetic testing.

**Interventions:**

The patient underwent a regimen of betahistine therapy combined with vestibular rehabilitation training.

**Outcomes:**

The patient exhibited clinical improvement and was discharged after 2 weeks.

**Lessons:**

Detailed neuro-otological and neuro-ophthalmological evaluations are helpful for the diagnosis of patients with suspected cerebellar ataxia.

## 1. Introduction

Spinocerebellar ataxia type 3 (SCA3) is the most common autosomal dominant inherited spinocerebellar disorder. This is caused by expansion of the CAG repeat in the ataxin 3 gene. In healthy individuals, this gene contains 12 to 44 CAG repeats.^[1]^ The repeat sequences of patients with SCA3 expand to 52 to 91, which leads to the aggregation of polyglutamine in the nuclei of brain neurons and brain damage without clinical manifestations.^[2]^

Clinical symptoms of SCA3 are characterized by progressive cerebellar ataxia. Other symptoms include dysarthria, oculomotor abnormalities, pyramidal tract signs, extrapyramidal system signs, and non-motor symptoms.^[3]^ The diversity of symptoms is a significant challenge in clinical diagnosis of SCA3.

In this study, we presented a case of a female Chinese patient who frequently sought treatment for dizziness. The patient was initially misdiagnosed with persistent postural-perceptual dizziness (PPPD) and received appropriate treatment. We discovered this patient with vestibular dysfunction, and her family members had similar symptoms. Finally, the patient was diagnosed with SCA3 by genetic testing.

## 2. Case report

A 55 years old woman from China experienced dizziness in 2017. The persistent symptoms of dizziness were exacerbated by complex visual stimuli. As the disease progressed, the patient slowly developed unstable gait. During the following years, she received relevant treatment for PPPD and finally came to our hospital because of ineffective therapy.

Neurological physical examination revealed that the 4 limbs had normal muscle strength and tone. Bilateral knee-jerk reflexes were more active than normal reflexes, but Babinski’s signs were negative. Patient with a broad-based gait and unstable tandem gait with a tendency towards the right. The Fukuda stepping test revealed a 90° turn to the left. Spontaneous nystagmus was negative, whereas slight horizontal gazing nystagmus was observed.

The patient’s history of illness was unremarkable. However, the patient’s mother, older sister, and younger brother showed similar symptoms. Her mother had severe symptoms and died at 60 years old. The older sister showed more severe dizziness and unsteadiness while walking, and the younger brother had only slight dizziness that did not affect daily life.

There were no abnormalities in the routine laboratory tests. Brain and cervical magnetic resonance imaging (MRI) revealed no obvious abnormalities. Hamilton Depression Scale and Hamilton Anxiety Scale scores were 19 and 20, respectively. The results of the video head impulse test (vHIT) and caloric test indicated that the peripheral vestibular function was impaired. An eye-tracking test suggested a central vestibular system dysfunction. In addition, the same vestibular function tests, both peripheral and central, suggested that the older sister had more severely impaired function and the son had normal function (Tables 1 and 2). Genetic testing of the patient revealed 10 and 68 CAG repeat sequences in the ataxin 3 gene.

**Table 1:** The results of slow-phase eye velocity from video head impulse test.

	Superior canal Right/Left	Horizontal canal Right/Left	Posterior canal Right/Left
Patient	0.58/0.61	0.62/0.59	0.73/0.59
Sister	0.47/0.31	0.27/0.32	0.37/0.31
Son	0.97/0.80	0.90/0.97	1.08/0.99

**Table 2:** Summary of examination results of caloric test and optokinetic test.

	Patient	Sister
Caloric test	Right 50 + Right 24 = 5°/s Left 50 + Left 24 = 3°/s	Right 50 + Right 24 = 5°/s Left 50 + Left 24 = 8°/s
	Bilateral horizontal semicircular canal function weaked	
Saccades	Upward: undershot Downward: overshoot Horizontal: normal	Upward: overshoot Downward: undershot Horizontal: normal
Optokinetic nystagmus	Bilateral normal	Bilateral abnormal
Eye tracking test	Both horizontal and vertical are type III curves	

During hospitalization, she received treatment with betahistine and rehabilitation training. The patient’s symptoms had significantly improved after 2 weeks of treatment.

## 3. Discussion

In this article, we describe a case of a patient with SCA3. The initial symptom of the patient was dizziness, followed by unsteady gait. In the early stages of illness, the patient frequently sought treatment for isolated dizziness. She was misdiagnosed with PPPD at another hospital because of normal brain MRI findings and emotional abnormalities. Long-term treatment did not improve her symptoms; therefore, she visited our hospital for assistance. In addition to routine laboratory tests and brain MRI, we performed neuro-otological and neuro-ophthalmological evaluations. Examinations of the physical and vestibular functions revealed impaired function of the peripheral and central vestibular systems. The typical family history and relevant examinations of family members have deepened our speculation regarding SCA3. We ultimately conducted genetic testing of the patient and genetic analysis supported the diagnosis of SCA3.

SCA3 is a neurodegenerative disease. The cerebellum and its connections, which play important roles in maintaining body balance, are the most common sites of degeneration in patients with SCA3.^[4]^ Neurodegenerative changes gradually deteriorate with the development of the illness, resulting in exacerbated symptoms.^[1]^

The definitive diagnosis of SCA3 primarily relies on genetic testing, but its use is still limited in clinical practice.^[5]^ Performing genetic testing in the absence of sufficient evidence is not only time-consuming but also adds to the patient’s economic burden.^[6]^ In clinical practice, neuro-otologic and neuro-ophthalmologic evaluations such as vHIT are simple and practicable.

The vestibulo-ocular reflex (VOR) maintains clear vision during rapid head movements. The vHIT is commonly used to measure compensatory slow-phase eye velocity (SPV) to assess the VOR.^[7]^ Previous studies have shown that the VOR gains of SCA3 decrease with abnormal catch-up saccades. Peripheral vestibular dysfunction in SCA3 was significantly reflected in the vHIT.^[8]^ In our case, the gains in the SPV of the patient and older sister were reduced. Older sister with more severe symptoms also showed lower SPV gains.

However, this patient may have been misdiagnosed with bilateral vestibulopathy (BVP) if we only considered clinical symptoms and peripheral vestibular damage. BVP is a chronic vestibular syndrome characterized by reduced function of the bilateral vestibular end-organs and/or their pathways.^[9]^ SCA3 not only exhibits peripheral vestibular dysfunction, but also central vestibular dysfunction. Examinations of the central vestibular system can help distinguish between SCA3 and BVP.

There is no specific treatment for SCA3 and disease-modifying treatments can delay disease progression. Therefore, early diagnosis is of great significance in improving the quality of life of SCA3 patients.^[10]^ The characteristic clinical manifestations of SCA3 may not be evident in the early stages. For patients with suspected SCA3, detailed medical history, physical examinations, and auxiliary examinations should be combined. Vestibular function examinations can provide a more comprehensive understanding and evaluation of cerebellar function to reduce the misdiagnosis of SCA3 patients. Overall, we hope to review the diagnostic process of this case to provide a more comprehensive understanding of SCA3 and provide assistance for future clinical diagnosis.

## Author contributions

**Writing – original draft:** Yisi Zhang.

**Writing – review & editing:** Xiaoguang Chen, Songbin Pan.
